# Immune-related hepatitis and hypophysitis are associated with superior survival in melanoma patients treated with combined ipilimumab and nivolumab

**DOI:** 10.1080/2162402X.2025.2543510

**Published:** 2025-08-08

**Authors:** Hifaa Al Remawi, Maria Lindén, Zhiyuan Zhao, Ankur Pandita, Anna Rudin, Lars Ny, Sara Bjursten, Max Levin

**Affiliations:** a Department of Oncology, Institute of Clinical Sciences, Sahlgrenska Academy, University of Gothenburg, Gothenburg, Sweden; b Department of Oncology, Sahlgrenska University Hospital, Gothenburg, Sweden; c Department of Molecular and Clinical Medicine/Wallenberg Laboratory, Institute of Medicine, Sahlgrenska Academy, University of Gothenburg, Gothenburg, Sweden; d Department of Rheumatology and Inflammation Research, Institute of Medicine, Sahlgrenska Academy, University of Gothenburg, Gothenburg, Sweden

**Keywords:** CTLA-4 inhibitor, immune-related adverse events, melanoma, PD-1 inhibitor, survival

## Abstract

Combination CTLA-4 (ipilimumab) and PD-1 (nivolumab) checkpoint inhibition (dual-ICI) improves survival in patients with advanced melanoma. However, many patients also experience immune-related adverse events (irAE) that require systemic treatment with corticosteroids. Corticosteroids dampen the anti-tumoral response and may impair survival. Here, we investigated the association between irAE and overall survival as well as exposure to corticosteroids and second line immunosuppressants in dual ICI-treated patients with advanced melanoma (*n* = 205). Patients with irAE (*n* = 113) had superior OS compared to patients with no irAE (*n* = 92). The survival benefit persisted after adjusting for immortal time bias. Regarding specific irAE, patients with colitis, hepatitis, rheumatic irAE, hypophysitis, and skin-related irAE had improved OS after adjusting for negative baseline factors. A survival benefit persisted for hypophysitis (*p* = 0.03) and hepatitis (*p* = 0.04) after adjusting for immortal time bias, whereas rheumatic (*p* = 0.05) and skin-related irAE (*p* = 0.06) where borderline significant. Hepatitis and colitis required higher doses of corticosteroids for longer times and more often second-line immunosuppression compared to other irAE. In conclusion, irAE are associated with superior OS in patients with advanced melanoma treated with dual ICI. Hepatitis and hypophysitis were most strongly associated with better survival outcomes. Studies investigating the mechanisms underlying hepatitis and hypophysitis may identify important response mechanisms.

## Introduction

Treatment with immune checkpoint inhibitors (ICI) has improved survival in different types of cancer. The main targets of ICI are the inhibitory immune checkpoints cytotoxic T-lymphocyte-associated antigen 4 (CTLA-4) and programmed cell death protein-1 (PD-1). The physiological role of CTLA-4 and PD-1 is to decrease immune activation in order to avoid autoimmune damage caused by an overly active immune system.^1,2^ On the other hand, the immune system protects against cancer because activated T lymphocytes continuously patrol and eliminate malignant cells. During tumorigenesis, CTLA-4 and PD-1 signaling de-activates cytotoxic T cells, allowing cancer cells to evade cell killing. Blocking CTLA-4 and/or PD-1 keeps the T cells active and effectively eliminates cancer cells in a large proportion of patients. ICI can be achieved via monotherapy with either anti-CTLA-4 antibodies, such as ipilimumab, or anti-PD-1/anti-PD-L1 antibodies, such as nivolumab, or dual therapy with a combination of these agents. In patients with advanced melanoma,^3^ the 10-year survival rate is higher in patients treated with combined ipilimumab and nivolumab (43%) compared to single nivolumab (39%) or single ipilimumab (19%).^4^ Importantly, dual ICI is more effective than PD-1 monotherapy in patients with renal clear cell carcinoma, lung cancer, and melanoma brain metastases.^3,5,6^

Given that immune checkpoints are crucial in the regulation of autoimmunity, ICI therapy increases the risk of autoimmune reactions.^7^ These autoimmune reactions, known as immune-related adverse events (irAE), can affect any organ and vary in severity from asymptomatic (grade 1) to fatal (grade 5), as graded by the Common Terminology Criteria for Adverse Events (CTCAE).^8^ The incidence and severity of irAE depend on the mechanism of checkpoint blockade and the treatment regime, i.e. single or dual ICI.^6^ Dual ICI yields a higher incidence and more severe irAE than monotherapy.^9^ The pattern of irAE varies; colitis, hepatitis, and hypophysitis have a stronger association with CTLA-4 blockade, while thyroid irAE and rheumatic irAE are more common with PD-1/PD-L1 blockade.^7^ Additionally, the more severe irAE induced by dual ICI requires treatment with high-dose corticosteroids, and other immunosuppressants, which may have a negative effect on the anti-tumoral response.^10–12^

Patients with melanoma treated with PD-1 inhibitors who develop irAE have better treatment outcomes than patients who do not develop irAE. However, in patients treated with CTLA-4 inhibitor monotherapy, there is no correlation between irAE and superior survival.^13,14^ In the case of dual ICI, the treatment regimen with the highest frequency of severe irAE and as well as the highest need for immunosuppressive treatments, the association between irAE and treatment outcome is largely unknown.

In this single-center retrospective study, we examined if there is a difference in overall survival (OS) between dual ICI-treated patients with advanced melanoma who develop irAE compared to those who do not. We then investigated if the survival benefit differs between patients with different types of high-grade irAE (i.e. irAE requiring intervention with corticosteroids or hormone replacement therapy), focusing on the most common irAE.

## Materials and methods

### Study design

This retrospective study included all consecutive patients diagnosed with unresectable stage III (M0) or stage IV cutaneous melanoma (M1a-d) treated with dual ICI at the Department of Oncology, Sahlgrenska University Hospital, Gothenburg, Sweden, between 26 March 2018 and 07 August 2023. Patients were identified from the oncology outpatient clinic register, and data were collected from electronic medical records. The cutoff date for data compilation was set to 15 August 2023, with data collected from 15 August 2023 to 30 October 2023. All patients over 18 years of age who received at least one dose of combined ipilimumab and nivolumab during the study period were included. Dual ICI was either ipilimumab 3 mg/kg and nivolumab 1 mg/kg or ipilimumab 1 mg/kg and nivolumab 3 mg/kg administered intravenously at the oncology outpatient clinic every third week for up to four doses, followed by maintenance nivolumab 480 mg for up to 24 months, starting six weeks after the last combined ipilimumab and nivolumab dose. The patients received treatment as long as it was well tolerated and the patient clinically benefited from treatment as assessed by the treating physician.

### Ethical considerations

The research protocol was approved by the Swedish Ethical Review (number 433–11). Requirement for informed consent was waived by the ethics committee because data are presented at group level and it is not possible to identify individual patients. The study adheres to the Declaration of Helsinki

### Data collection

The following baseline characteristics were collected: Eastern Cooperative Oncology Group (ECOG) performance status, sex, age, lactate dehydrogenase (LDH) in blood, baseline c-reactive protein in blood (CRP), BRAF mutation status, body mass index (BMI), disease stage (M0, M1a, M1b, M1c, or M1d), metastatic sites at treatment start, and whether patients have received any systemic treatment prior to dual ICI. For LDH, the upper limit of normal (ULN) was defined as 3.4 µkat/L. Patients with missing data entries for a certain baseline characteristic, e.g. unknown BMI or baseline CRP, were excluded from the statistical analysis considering that specific variable. Other relevant information, such as treatment start date, death date, number of ipilimumab + nivolumab doses, number of maintenance nivolumab doses received, and reason for treatment discontinuation, was also collected.

The occurrence and severity of irAE were assessed by the treating physician or nurse and graded according to the CTCAE v5.0^8^ when applicable and documented in the medical records. Medical intervention was initiated according to the European Society for Medical Oncology guidelines for irAE.^15^ CNS irAE (encephalitis/meningitis/myelitis) were defined according to criteria by Guidon et al.^16^ In the survival analysis, irAE that required either systemic immunosuppressive treatment or endocrine replacement therapy were included. Depending on the type of irAE, this corresponds to irAE grade 2–4 according to the CTCAE. For each type of irAE, the following parameters were collected: start dose of corticosteroids, additional immunosuppression (besides corticosteroids), and duration of immunosuppressive treatment. For the treatment of endocrine irAE, the start of endocrine replacement therapy was registered.

### Outcomes and statistical analyses

The primary endpoint was OS, defined as the number of days from treatment initiation to death or the censoring date. Differences in median OS were visualized using Kaplan-Meier curves and assessed through hazard ratios (HR) of death, calculated using the Cox proportional hazards model. The log-rank (Mantel-Cox) test was used to determine the statistical significance of survival differences between groups in the initial univariate analysis.

Baseline characteristics were categorized into groups to facilitate the analysis. For baseline analysis, we included sex (male vs. female), age (<65 vs. ≥65 years), BMI (<25 vs. ≥25), ECOG performance status (0–1 vs. 2–4), stage (M0-M1b vs. M1c-M1d), metastatic sites (<3 vs. ≥3), baseline LDH (<2 × ULN vs. ≥2 × ULN), baseline CRP (<5 vs. ≥5 mg/L), previous treatment (yes or no) and activating BRAF mutation (yes or no). The cutoff points of these variables were chosen based on previous studies reporting baseline characteristics,^3,17,18^ except for CRP, where the lab reference interval was used to determine the cutoff point.

The analysis of irAE started by dividing the patient cohort into two groups: those with any irAE and those with no irAE. HR was calculated and compared between these two groups. Subsequently, patients with specific irAE were compared to the ‘no irAE’ cohort using univariate Cox regression followed by multivariate Cox regression to adjust HR for negative prognostic baseline factors. Finally, time-dependent Cox regression was applied to account for immortal time bias. In this analysis, irAE status was treated as a time-dependent variable, allowing patients to transition from the ‘no irAE’ to the ‘irAE’ group upon developing an irAE. The HR was then calculated using the Cox proportional hazards model.

The validity of the Cox proportional hazards assumption was assessed using the scaled Schoenfeld residuals method. When the proportional hazards assumption was violated, as indicated by a significant test result (p-value < 0.05), an interaction term between the covariate and time was added to adjust for changes in HR over time. Baseline HRs were then reported, along with a description of how HR changes over time. 95% confidence intervals (CI) were calculated and reported for median OS and HR, and p-values of <0.05 were considered statistically significant. Adjusted p-values were calculated using the Wald test. R version 4.4.2 and GraphPad Prism 10.4.0 were used for the statistical analyses. All statistical analyses were performed with assistance from professional biostatisticians.

## Results

### Baseline characteristics

From the medical records, 205 patients were identified. Patient characteristics at baseline are presented in Table 1. A total of 154 out of 205 patients (75.1%) had received systemic anti-tumoral treatment before combined ipilimumab and nivolumab. Of these, 116 (75.3%) had been treated with single PD-1 or CTLA-4 inhibitors at some point before dual ICI.

**Table 1.** Baseline patient characteristics.

Characteristics		All patients *n* = 205(%)	No irAE *n* = 92(%)	irAE *n* = 113(%)
Sex	Female	81 (39.5)	34 (37)	47 (41.6)
	Male	124 (60.5)	58 (63)	66 (58.4)
Age	Median	66	67	64
	Range	27–88	27–87	29–88
	≤65	98 (47.8)	41 (44.6)	57 (50.4)
	>65	107 (52.2)	51 (55.4)	56 (49.6)
BMI	≤25	79 (38.5)	36 (39.1)	43 (38.1)
	>25	111 (54.1)	48 (52.2)	63 (55.8)
	Unknown	15 (7.3)	8 (8.6)	7 (6.2)
PS (ECOG)	PS 0–1	188 (91.7)	80 (87)	108 (95.6)
	PS 2–4	17 (8.3)	12 (13)	5 (4.4)
Stage	M0	8 (3.9)	4 (4.3)	4 (3.5)
	M1a	24 (11.7)	11 (12)	13 (11.5)
	M1b	25 (12.2)	6 (6.5)	19 (16.8)
	M1c	74 (36.1)	36 (39.1)	38 (33.6)
	M1d	71 (34.6)	33 (35.9)	38 (33.6)
	Unknown	3 (1.5)	2 (2.2)	1 (0.8)
Metastatic sites	≤3	104 (50.7)	43 (46.7)	61 (54)
	>3	101 (49.3)	49 (53.3)	52 (46)
Baseline LDH	≤2xULN	164 (80.0)	68 (74)	96 (85)
	>2xULN	40 (19.5)	24 (26)	16 (14.2)
	Unknown	1 (0.5)	0	1 (0.8)
Baseline CRP	≤5	83 (40.5)	30 (32.6)	53 (47)
	>5	98 (47.8)	51 (55.4)	47 (41.5)
	Unknown	24 (11.7)	11 (12)	13 (11.5)
BRAF mutation	No	105 (51.2)	48 (52.2)	57 (50.4)
	Yes	92 (44.9)	41(44.6)	51 (45.1)
	Unknown	8 (3.9)	3 (3.4)	5 (4.4)
Previous treatment Type of previous treatment	No	51 (24.9)	19 (20.7)	32 (28.3)
	Yes Anti-PD1 Anti-CTLA4 BRAFi Other	154 (75.1) 105 (51.1) 11 (5.4) 56 (27.3) 16 (7.8)	73 (79.3) 47 (51.1) 6 (6.5) 29 (31.5) 6 (6.5)	81 (71.7) 58 (51.3) 5 (4.4) 27(23.9) 10 (8.8)

” >BMI: body mass index, PS (ECOG): performance status (Eastern Cooperative Oncology Group), LDH: lactate dehydrogenase, ULN: upper limit of normal (3.4 µkat/L), CRP: C-reactive protein, BRAFi: BRAF inhibitors.

### Survival outcomes in patients with different baseline characteristics

**Figure 1.**
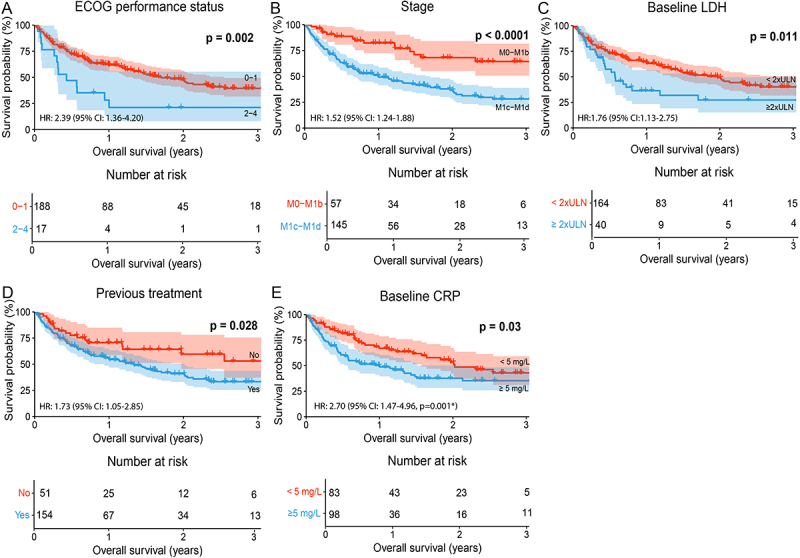
Baseline characteristics associated with impaired overall survival: A. ECOG performance status ≥2 (*p* = 0.002), B. clinical stage M1c/M1d (*p* < 0.0001), C. baseline LDH ≥ 2xULN (*p* = 0.011), D. previous treatment (*p* = 0.028) and F. baseline CRP ≥5 mg/ml (*p* = 0.03) are associated with impaired OS as illustrated in Kaplan-Meier curves and hazard ratios (HR). HR were calculated using a univariate Cox proportional hazards model. HR > 1 indicates a higher risk of death. P-values are calculated with the log-rank test, where *p* < 0.05 was considered statistically significant. Confidence intervals (CI) for median survival and HR were set to 95%.

The following baseline characteristics were associated with decreased median OS: ECOG performance status ≥2 (HR: 2.39, 95% CI: 1.36–4.20, *p* = 0.002, Figure 1A), stage M1c/M1d (HR: 3.04, 95% CI: 1.76–5.25, *p* < 0.001, Figure 1B), baseline LDH level ≥2 × ULN (HR: 1.76, 95% CI: 1.13–2.75, *p* = 0.011, Figure 1C), and previous systemic anti-tumoral treatment (HR: 1.73. 95% CI: 1.05–2.85, *p* = 0.028, Figure 1D). Patients with baseline CRP ≥5 mg/L had decreased OS (Figure 1E, *p* = 0.03) and an initially higher risk of death (HR: 2.70, 95% CI: 1.47–4.96, *p* = 0.001), with the HR decreasing over time. Sex (*p* = 0.73), age (*p* = 0.17), BMI (*p* = 0.16), BRAF mutation status (*p* = 0.46), and metastatic sites (*p* = 0.3) had no statistically significant impact on OS (Figure S1).

*:The proportional hazards assumption was violated for baseline CRP, and HR was therefore adjusted using time-varying covariates. The presented HR represents the baseline HR, which decreased over time. The adjusted p-value was calculated using the Wald test. ECOG: Eastern Cooperative Oncology Group, LDH: lactate dehydrogenase, ULN: upper limit of normal (3.4 µkat/L), CRP: C-reactive protein.

Patients with missing data on BMI (*n* = 15), stage (*n* = 3), baseline LDH (*n* = 1), baseline CRP (*n* = 24), and those who had unknown BRAF mutation status (*n* = 8) were excluded from analyses examining that specific variable.

### Immune-related adverse events (irAE)

113 out of 205 patients (55.1%) experienced any irAE at some point during the study period. Baseline characteristics of patients with or without irAE are shown in Table 1. 38 patients had more than one irAE during the study period, with 15 patients developing simultaneous irAE (Figure S2).

**Figure 2.**
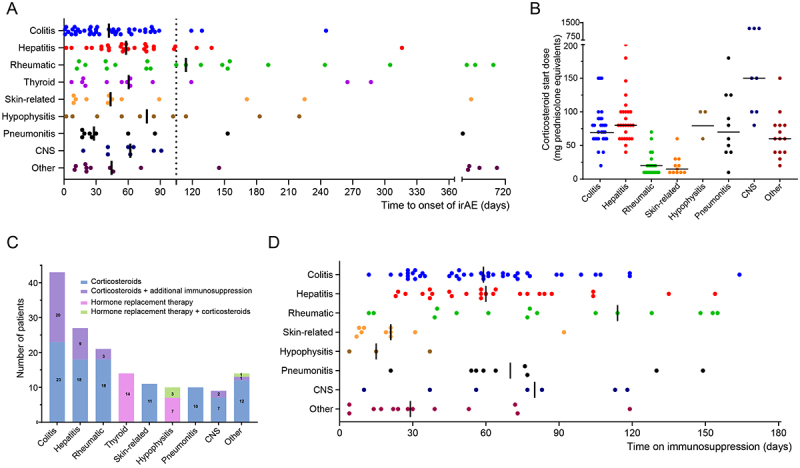
Frequency, onset and management of irAE. A. Time to irAE onset for patients who developed irAE; the dashed line marks 15 weeks (105 days) from treatment start. B. Corticosteroids start dose (mg, prednisolone equivalents) to manage irAE. The black lines represent the median start dose. C. Number of patients in different irAE groups who needed additional immunosuppressive treatment besides corticosteroids to manage irAE. D. Time on immunosuppression for different irAE. The black lines represent the median time on immunosuppression. irAE: immune-related adverse events, CNS: central nervous system.

The most frequently observed irAE were colitis (21%, 43/205), hepatitis (13.1%, 27/205), rheumatic irAE (10.2%, 21/205), thyroiditis (6.8%, 14/205), skin-related irAE (5.4%, 11/205), hypophysitis (4.9%, 10/05), pneumonitis (4.9%, 10/205) and CNS-irAE (4.4%, 9/205). 14 patients developed other irAE, described in Table S1. 95 of 113 irAE (84.1%) first occurred within 105 days after the first dose of combined ipilimumab and nivolumab (Figure 2A). Time to onset varied between different irAE; pneumonitis tended to occur early (median: 28 days, IQR:19–119), and rheumatic irAE tended to occur late (median: 114 days, IQR:44–218).

Patients with irAE were treated with corticoteroids or HRT (for endocrine irAE). Colitis, hepatitis, pneumonitis and CNS irAE required a higher dose of corticosteroids than rheumatic and skin-related irAE (Figure 2B). 35 irAE required additional immunosuppressive treatment besides corticosteroids to resolve. 46.5% (20/43) of patients with colitis and 33.3% (9/27) of patients with hepatitis required additional immunosuppressive treatment (Figure 2C and Table S2). Most irAE resolved within 180 days of immunosuppression as illustrerated in Figure 2D.

### Survival outcomes in patients experiencing immune-related adverse events (irAE)

**Figure 3.**
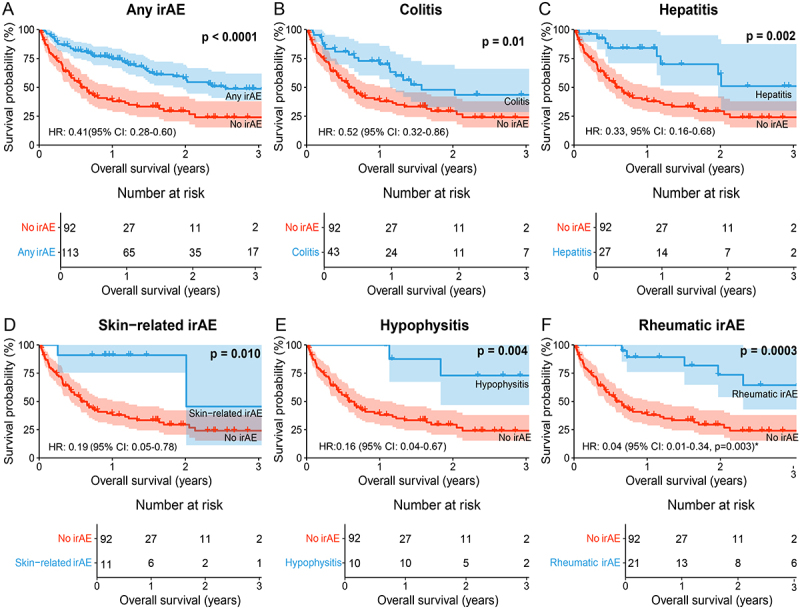
OS in patients with or without irAE (A, *p* < 0.0001). Specifically, patients with colitis (B, *p* = 0.01), hepatitis (C, *p* = 0.0018), skin-related irAE (D, *p* = 0.0099), hypophysitis (E, *p* = 0.004), and rheumatic irAE (F, *p* = 0.00028), had superior OS compared to patients with no irAE during the study period. Hazard ratios (HR) were calculated using a univariate Cox proportional hazards model. HR < 1 indicates a lower risk of death. P-values are calculated with the log-rank test, where *p* < 0.05 was considered statistically significant. Confidence intervals (CI) for median survival and HR were set to 95%. irAE: immune-related adverse events. *: the proportional hazards assumption was violated for rheumatic irAE, and HR was therefore adjusted using time-varying covariates. The presented HR represents the baseline HR, which increased over time. The adjusted p-value was calculated using the Wald test. irAE: immune-related adverse events.

Patients with any irAE had superior median OS and a lower risk of death compared to patients with no irAE (HR: 0.41, 95% CI: 0.28–0.60, *p* < 0.0001) (Figure 3A). Patients with irAE following dual ICI treatment had superior overall survival (OS), regardless of whether they had previously received checkpoint inhibitor monotherapy (Figure S3). In the analysis of survival outcomes for specific irAE, patients with colitis (HR: 0.52, 95% CI: 0.32–0.86, *p* = 0.01, Figure 3B), hepatitis (HR: 0.33, 95% CI: 0.16–0.68, *p* = 0.002, Figure 3C), skin-related irAE (HR: 0.19, 95% CI: 0.05–0.78, *p* = 0.010, Figure 3D), and hypophysitis (HR: 0.16, 95% CI: 0.04–0.67, *p* = 0.004, Figure 3E) had a statistically significantly longer median OS and a lower risk of death compared to patients with no irAE. Patients who developed rheumatic irAE had superior OS as shown in Figure 3F (*p* = 0.003). The proportional hazards assumption was violated for rheumatic irAE, meaning that the HR varied over time. At baseline, patients with rheumatic irAE had a lower risk of death (HR: 0.04, 95% CI: 0.01–0.34, *p* = 0.003). However, this protective effect diminished over time, with the HR increasing as follow-up continued. There was no statistically significant difference in OS or risk of death in patients experiencing pneumonitis (HR: 0.53, 95% CI: 0.21–1.33, *p* = 0.17), CNS-related irAE (HR: 0.47, 95% CI: 0.17–1.30, *p* = 0.14), or thyroid irAE (*p* = 0.17) (Figure S4). The proportional hazards assumption was violated for thyroid irAE, and the HR at baseline indicated a lower risk of death (HR: 0.21, 95% CI: 0.06–0.80, *p* = 0.022). However, this survival benefit diminished over time, with the HR increasing throughout the study period.

**Figure 4.**
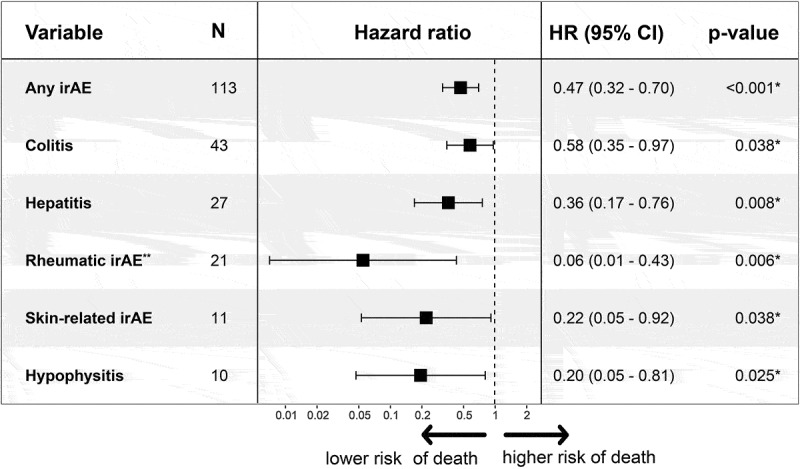
Multivariate Cox regression to adjust for negative prognostic baseline factors. Multivariate COX regression involving specific irAE associated with improved overall survival adjusting for ECOG performance status ≥ 2, baseline LDH ≥ 2xULN, and stage M1c-M1d. The survival benefit persisted for any irAE (HR: 0.47, *p* < 0.001), colitis (HR: 0.58, *p* = 0.038), hepatitis (HR: 0.36, *p* = 0.008), rheumatic irAE (adjusted HR: 0.06, *p* = 0.006), skin-related irAE (HR: 0.22, *p* = 0.038), and hypophysitis (HR: 0.20, *p* = 0.025). P-values are calculated with the Wald test. Confidence intervals (CI) for HR were set to 95% and *p* < 0.05 was considered statistically significant and marked with *. **: the proportional hazards assumption was violated for rheumatic irAE, and HR was therefore adjusted using time-varying covariates. The presented HR represents the baseline HR, which increased over time. irAE: immune-related adverse events. ECOG: Eastern Cooperative Oncology group, LDH: lactate dehydrogenase, ULN: upper limit of normal (3.4 µkat/L).

Next, adjustment was made for the negative prognostic factors of worse ECOG performance status, elevated baseline LDH, and more advanced cancer stage. The lower risk of death persisted for patients who experienced any irAE (HR: 0.47, 95% CI: 0.32–0.7, *p* < 0.001), colitis (HR: 0.58, 95% CI: 0.35–0.97, *p* < 0.038), hepatitis (HR: 0.36, 95% CI: 0.17–0.76, *p* = 0.008), skin-related irAE (HR: 0.22, 95% CI: 0.05–0.92, *p* = 0.038) and hypophysitis (HR: 0.20, 95% CI: 0.05–0.81, *p* = 0.025) after adjusting for these factors (Figure 4). For patients with rheumatic irAE, the low risk of death persisted in the adjusted analysis at baseline (HR: 0.06, 95% CI: 0.01–0.43, *p* = 0.006), but the risk increased over time.

**Figure 5.**
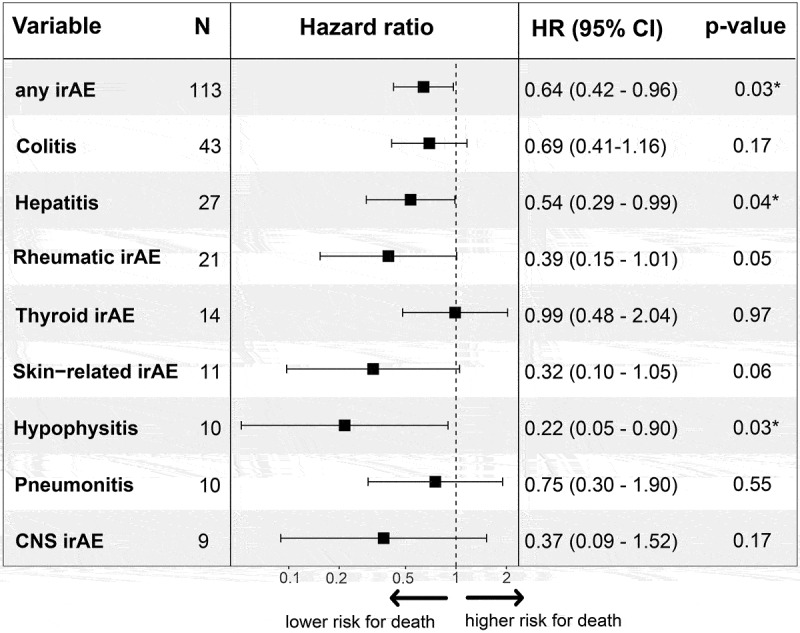
Time-dependent Cox regression to adjust for immortal time bias. Hepatitis (HR: 0.54, *p* = 0.04) and hypophysitis (HR: 0.22, *p* = 0.03) were associated with a significantly lower risk of death. Rheumatic irAE and skin-related irAE had a slightly lower risk of death, albeit not statistically significant (*p* = 0.05 and 0.06, respectively). No significant impact on the risk of death was observed in patients with colitis, thyroid irAE, pneumonitis, and CNS irAE compared to patients who did not develop any irAE during the study period. P-values were calculated using the Wald test, where *p* < 0.05 was considered statistically significant and marked with *. CI: confidence interval. irAE: immune-related adverse events. CNS: central nervous system.

Finally, time-dependent Cox regression was used to adjust for immortal time bias. The results are shown in a Forest plot in Figure 5. Patients who developed any irAE had a lower risk of death compared to patients who did not develop any irAE (HR: 0.64, 95% CI: 0.42–0.96, *p* = 0.03). Hepatitis (HR: 0.54, 95% CI: 0.29–0.99, *p* = 0.04) and hypophysitis (HR: 0.22, 95% CI: 0.05–0.90, *p* = 0.03) were associated with a statistically significant lower risk of death. Patients with skin-related irAE (HR: 0.32, 95% CI: 0.1–1.05, *p* = 0.06) and rheumatic irAE (HR: 0.39, 95% CI: 0.15–1.01, *p* = 0.05) had a borderline significant lower risk of death. Colitis (*p* = 0.2), thyroid (*p* = 0.97), CNS irAE (*p* = 0.17), and pneumonitis (*p* = 0.6) were not associated with a lower risk of death.

### irAE management and effect on overall survival

**Figure 6.**
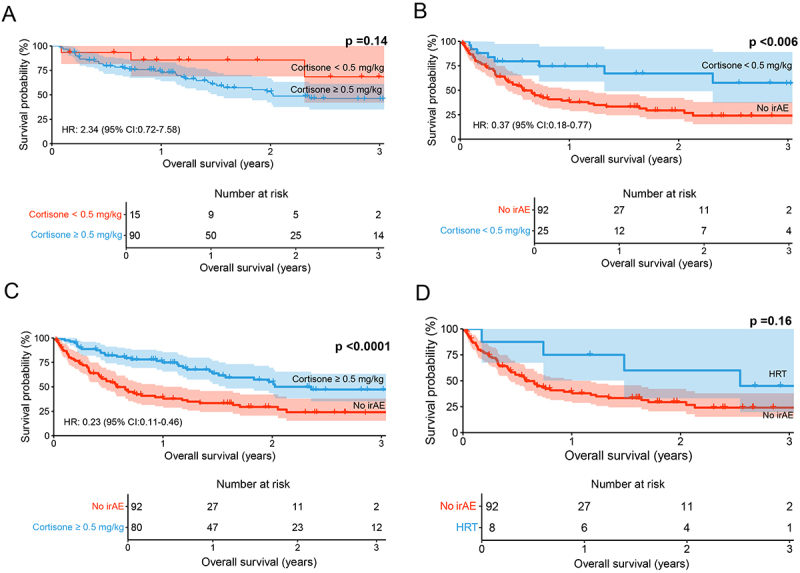
The impact of irAE management on OS. A. No statistically significant difference in OS was observed in patients receiving an initial cortisone dose > 0.5 mg/kg compared to patients who received ≤0.5 mg/kg (*p* = 0.14). B. Patients treated with cortisone > 0.5 mg/kg had superior OS compared to patients with no irAE (*p* < 0.0001). C. Patients treated with cortisone ≤0.5 mg/kg had superior OS compared to patients with no irAE (*p* = 0.006). D. No statistically significant difference in OS was observed in patients receiving hormone replacement therapy (HRT) compared to patients with no irAE (*p* = 0.16). P-values are calculated with the log-rank test, where *p* < 0.05 was considered statistically significant. irAE: immune-related adverse events, HRT: hormone-replacement therapy.

There was no significant difference in OS between patients who received initial cortisone doses >0.5 mg/kg mg and those who received initial cortisone doses ≤0.5 mg/kg (HR: 2.34, 95% CI: 0.72–7.58, *p* = 0.14, Figure 6A). However, patients who were treated with cortisone doses >0.5 mg/kg (HR: 0.23, 95% CI: 0.11–0.46, *p* < 0.0001, Figure 6B) and those who were treated with doses ≤0.5 mg/kg (HR: 0.37, 95% CI: 0.18–0.77, *p* = 0.0038, Figure 6C) had superior survival and lower risk of death compared to patients who had not developed any irAE. Patients who only received HRT during the study period did not have a significant difference in OS compared to patients with no irAE (HR: 0.52, 95% CI: 0.20–1.31, *p* = 0.16, Figure 6D).

## Discussion

This study demonstrates that irAE are a clinical marker for superior OS in patients with advanced melanoma treated with combined CTLA-4 and PD-1 inhibition. Specifically, in patients who developed hepatitis and hypophysitis, the association remained significant after adjusting for immortal time bias. The frequency of irAE in our cohort was 55.1%, in line with previous studies.^3,9,19^ The types, severity, management and early onset of irAE in this study are consistent with what has been reported in patients treated with combined PD-1 and CTLA-4 inhibition for melanoma and other cancers.^3,7,20^

The observation that hepatitis was strongly associated with a survival benefit in melanoma patients treated with double ICI is interesting. Hepatitis is more common in patients treated with dual ICI than in patients treated with single PD-1 blockade.^3^ This suggests that CTLA-4 inhibition is critical to induce hepatitis. One explanation could be that CTLA-4 blockade induces stronger activation of CD4+ T cells than PD-1 blockade (which mainly activates CD8+ cells)^21^ and CD4+ T cells may promote several irAE, including hepatitis.^22–24^ In support of this potential mechanism, patients with genetic CTLA-4 deficiencies have increased proportions of activated CD4+ T cells both in the blood and in the liver.^25–27^ Thus, infiltration of immune cells into the liver, due to either genetic CTLA-4 deficiency or CTLA-4 inhibitors, may promote hepatitis. We speculate that the superior response in patients with hepatitis may also be explained by increased activation of CD4+ T cells. Identifying the exact phenotype of these cells could provide important clues on the shared immune mechanisms behind liver toxicity and survival.

Patients with hepatitis had the highest starting dose of corticosteroids (along with patients with CNS irAE), the second highest need of second line immunosuppression (along with colitis) and the longest time on corticosteroid treatment (along with rheumatic, pneumonitis and CNS irAE). Consequently, the observed survival benefit in the group of patients with hepatitis was evident despite the high cumulative dose of immunosuppressive corticosteroids and frequent need of second line treatment with broadly immunosuppressive mycophenolate mofetil. A recent combined analysis of six clinical trials of dual ICI-treated patients showed that a high peak dose of corticosteroids was associated with worse survival across tumor types, whereas a high cumulative dose was not.^11^ Other studies indicate that early administration of high doses of corticosteroids to manage irAE is associated with poorer OS.^10,12^ As shown in this study, hepatitis is strongly associated with better survival. Positive immune activation in patients with hepatitis may partly compensate for the potentially negative effects of immunosuppressive corticosteroids on survival. In support of this notion, the addition of high-dose corticosteroids to HRT in patients with ipilimumab-induced hypophysitis is associated with worse survival.^28^ With more specific treatment than currently used broadly immunosuppressive high-dose corticosteroids, survival may be further improved in patients with hepatitis. However, in order to develop such targeted treatment, it is necessary to define the exact immune mechanisms behind dual ICI-induced hepatitis.

In addition to hepatitis, hypophysitis was strongly associated with superior OS. Similar to hepatitis, hypophysitis is common in patients treated with combined CTLA-4 and PD-1 blockade but rare during single PD-1 blockade.^3^ In contrast to hepatitis, the high incidence of hypophysitis can probably not be explained by CTLA-4 blockade per se because hypophysitis is rare in patients with genetic CTLA-4 deficiency.^25^ A more likely explanation is that subsets of cells in the pituitary gland express CTLA-4. Ipilimumab binds directly to CTLA-4 on these cells which promotes hypophysitis via complement-induced inflammation.^25,29^ Another plausible contributing factor to hypophysitis is increased immune cell infiltration into the brain (including the pituitary gland), a characteristic of genetic CTLA-4 deficiency.^26^ This could also explain the relatively high incidence of CNS irAE in our study, consistent with previous reports that CNS irAE are more common in patients receiving treatment with combined CTLA-4 and PD-1 blockade compared to single PD-1 blockade.^30,31^

In agreement with this study, previous reports have reported a survival benefit in patients with ipilimumab-induced hypophysitis. In one retrospective study, melanoma patients with ipilimumab-induced hypophysitis had superior OS than those who did not.^28^ In another study, hypophysitis was associated with superior survival in patients with various cancers treated with single ipilimumab (*n* = 120) or combined ipilimumab and nivolumab (*n* = 50). The association with superior survival was significant in the univariate analysis but not in the multivariate analysis adjusting for age, sex, and type of cancer.^32^ Moreover, an additional investigation demonstrated superior survival in patients developing ICI-induced hypophysitis following PD-1 (6%) or CTLA 4-blockade (24%) in both melanoma and non-small cell lung cancer.^33^ In summary, available evidence strongly suggests superior survival in patients experiencing ICI-induced-hypophysitis. In this study, we showed that this survival benefit persists after adjusting for negative baseline characteristics and immortal time bias. However, the shared immune mechanism behind hypophysitis and survival remains to be elucidated.

As expected, colitis was the most common irAE in our study.^3^ Colitis was also associated with superior OS and a lower risk of death. Similar to hepatitis, colitis required high doses of corticosteroids and often second-line immunosuppression (most often with TNF-inhibition). The survival benefit persisted after adjusting for negative prognostics baseline factors but not when adjusting for immortal time bias. Abu-Sbeih et al. have shown that immune-induced colitis was associated with longer OS in melanoma patients receiving any immunotherapy regimen.^34^ The observed survival benefit persisted after adjusting for negative prognostic factors and immortal time bias. Another study concluded that moderate colitis correlated with improved OS in patients treated with anti-CTLA4 but not with dual ICI. While our study affirms colitis as an independent factor of survival, we did not find colitis to be statistically significant in the time-dependent Cox regression. Notably, our study included 43 patients with higher-grade colitis, compared to 173 patients with any grade of colitis in the study by Abu-Sbeih et al.^34^ Whether our findings are a reflection of the smaller sample size or an actual effect of immortal time bias remains to be examined.^35^ In summary, available studies suggest an association between colitis and survival in patients treated with combined ICI, but this finding needs to be further validated.

Approximately 6% of patients in our study cohort developed skin-related irAE that required treatment with oral corticosteroids. Skin-related irAE were linked to superior OS in both univariate and multivariate analyses. The protective effect of skin-related irAE diminished, however, after adjusting for immortal time bias (*p* = 0.06). In contrast to most other types of irAE, OS has been extensively studied in relation to skin-related irAE with several studies showing that patients with skin-related irAE have a superior OS compared to patients with no skin-related irAE.^36,37^ While most of these studies have included patients with milder grades of skin-related irAE, typically treated with topical steroids, our study demonstrates a survival benefit extending to patients with skin-related irAE treated with oral prednisolone.

In contrast to previously discussed irAE, rheumatic irAE are more strongly associated with PD-1 inhibition. Supporting the role of PD-1 in rheumatic irAE, very early-onset arthritis has been reported in genetic PD-1 deficiency.^25^ In our cohort, 12 of the 21 patients experienced rheumatic irAE during the maintenance nivolumab phase, possibly reflecting higher dependency on PD-1 inhibition than other irAE in this study. Interestingly, the risk of death for patients with rheumatic irAE varied over time. The initially lower HR could be attributed to an early survival benefit due to immortal time bias that diminishes with time. The survival benefit was not significant after adjusting for immortal time bias (*p* = 0.05). In contrast to our results from patients treated with dual ICI, the association between rheumatic irAE with superior survival is clearer in patients treated with PD-1 inhibition. A meta-analysis by Maeda et al. showed rheumatic irAE to be associated with improved OS.^38^ Similar to rheumatic irAE, several studies show that thyroid irAE are associated with improved outcomes following PD-1 inhibition.^39–41^ A meta-analysis by Cheung et al. showed a superior OS for patients with thyroid irAE in both dual ICI and anti-PD-1 therapy.^42^ Our study shows significantly longer survival in patients with rheumatic irAE in the univariate analysis and when adjusting for prognostic baseline factors. Our data suggest (*p* = 0.05 when adjusting for immortal time bias) that rheumatic irAE are also associated with improved survival in patients treated with dual ICI.

Increased baseline CRP was a negative prognostic factor in this study in agreement with results from the Checkmate067 trial.^43^ Increased CRP is a marker of systemic inflammation and may indicate a higher tumor burden. The varying HR reflects the dynamic nature of systemic inflammation during treatment, which is not captured by a single baseline measurement. Consequently, these findings should be interpreted with caution. Moreover, elevated CRP could be due to multiple factors, such as infections and recent surgery. In line with most studies, elevated baseline LDH, worse ECOG performance status, and higher clinical stage were negative prognostic factors in our cohort.^9,19,44^

The major limitations of this study are the retrospective single-center design and that we only included melanoma patients. This may limit the generalizability of the results to other populations receiving dual ICI. Nevertheless, the single center/single cancer design is also an advantage since all irAE were managed by a small group of physicians and nurses according to ESMO guidelines, providing consistent care, and the intervals for blood samples were identical. This ensures that data regarding the classification and treatment of irAE are homogenous. Importantly, several irAE are objectively diagnosed as they present with distinct laboratory abnormalities, such as hepatitis (increased AST/ALT), thyroid irAE (increased TSH and low thyroxin), and hypophysitis (low cortisol, and other hormone abnormalities depending on the affected axis).

In conclusion, irAE are associated with superior OS in patients with advanced melanoma treated with ipilimumab and nivolumab and could serve as a clinical marker for treatment efficacy. Hepatitis and hypophysitis were most strongly associated with better survival outcomes. Studies investigating the mechanisms underlying these specific irAE could provide insight into important response mechanisms.

## Supplementary Material

Supplemental Material

## Data Availability

Anonymized data will be made available upon request to the corresponding author. Proposals will be reviewed and approved by the authors based on scientific merit.
