# Supplementary material for: Immune-related hepatitis and hypophysitis are associated with superior survival in melanoma patients treated with combined ipilimumab and nivolumab
Source: Oncoimmunology. 2025 Aug 8;14(1):2543510. doi: 10.1080/2162402X.2025.2543510 (PMC12952734; doi:10.1080/2162402X.2025.2543510)
Supplement: Supplemental Material [file KONI_A_2543510_SM6212.zip › New folder/Figure captions Supplementary figures.docx]

**Figure captions – supplementary figures**

**Figure S1: Baseline characteristics with no statistically significant association with overall survival**

Sex (A), BMI (B), age (C), BRAF mutation (D), and metastatic sites (E). P-values are calculated with the log-rank test. Hazard ratios (HR) of death are calculated using univariate Cox regression. HR<1 indicates a lower risk of death, and HR>1 indicates a higher risk of death. Confidence intervals (CI) are set to 95%. BMI: body mass index.

**Figure S2: Multi-irAE**

Pie-chart depicting how many specific irAE patients experienced during the study period (A). Upset plot depicting which irAE that occurred simultaneously (B).

**Figure S3: Dual ICI induced irAE and association with survival in patients with or without previous ICI monotherapy**

Overall survival in dual checkpoint inhibitor(combined PD-1 and CTLA-4 inhibition) treated patients with (A) or without (B) previous systemic treatment with single checkpoint inhibition (anti-PD-1 or anti-CTLA-4) . P- values are calculated with the log-rank test. Hazard ratios (HR) of death are calculated using univariate Cox regression. HR<1 indicates a lower risk of death, and HR>1 indicates a higher risk of death. Confidence intervals (CI) are set to 95%.

**Figure S4: irAE with no statistically significant association with overall survival**

Thyroid irAE (A), pneumonitis (B), and CNS irAE (C) were not associated with survival. P- values are calculated with the log-rank test. Hazard ratios (HR) of death are calculated using univariate Cox regression. HR<1 indicates a lower risk of death, and HR>1 indicates a higher risk of death. Confidence intervals (CI) are set to 95%. *: The proportional hazards assumption was violated for thyroid irAE, and HR was therefore adjusted using time-varying covariates. The presented HR represents the baseline HR, which increased over time. The adjusted p-value was calculated using the Wald test. irAE: immune-related adverse events, CNS: central nervous system.
