# Supplementary material for: Immune-related hepatitis and hypophysitis are associated with superior survival in melanoma patients treated with combined ipilimumab and nivolumab
Source: Oncoimmunology. 2025 Aug 8;14(1):2543510. doi: 10.1080/2162402X.2025.2543510 (PMC12952734; doi:10.1080/2162402X.2025.2543510)
Supplement: Supplemental Material [file KONI_A_2543510_SM6212.zip › New folder/Supplementary tables.docx]

# **Table S1: “Other irAE” in the patient cohort**

| **Type** | **No. (% of all**  **patients)** |
| --- | --- |
| **Nephritis** | 4(1.9) |
| **Other GI irAE** | 3(1.5) |
| **General inflammatory response** | 2(0.9) |
| **Neuropathy** | 2(0.9) |
| **Uveitis** | 1(0.4) |
| **Adrenal insufficiency** | 1 (0.4) |
| **Dry mouth** | 1(0.4) |

# **Table S2: A summary of the irAE cohort**

| **Specific irAE** | **No. (% of all patients)** | **Median** time (days) to onset  (IQR) | **Median** corticosteroids **start dose**  **(mg**, prednisolone equivalent**)** | **Median time (days) on immunosuppression**  (IQR) | **No. who received additional treatment (%)** | **Type of additional treatment (No.)** |
| --- | --- | --- | --- | --- | --- | --- |
| **Colitis, all** | 43(21.0) | 42 (15-71) | 70 | 59 (33-77) | 20 (46.5) | Infliximab (17) |
| **Grade 3-4** | 32 (15.6) |  |  |  |  | Infliximab + Vedolizumab (2) |
|  |  |  |  |  |  | Infliximab + Vedolizumab + MMF (1) |
| **Hepatitis, all** | 27(13.2) | 58 (41-84) | 80 | 60 (38-104) | 9 (33.3) | MMF (9) |
| **Grade 3-4** | 21 (10.2) |  |  |  |  |  |
| **Rheumatic irAE** | 21(10.2) | 114 (44-218) | 20 | 114 (55-218*) | 3 (14.3) | Methotrexate (1) |
|  |  |  |  |  |  | Methotrexate + TNF-α inhibitor (1) |
|  |  |  |  |  |  | TNF-α inhibitor (1) |
| **Thyroid irAE** | 14(6.8) | 61 (19-192) | ** | ** | 0 |  |
| **Hypophysitis** | 10(4.9) | 78 (20-149) | 100*** | 15 (4-37) *** | 3 (30) | Corticosteroids (3) |
| **Pneumonitis** | 10(4.9) | 28 (19-119) | 70 | 70 (56-135) | 0 |  |
| **Skin-related irAE** | 11(5.4) | 44 (11-171) | 15 | 21 (9-31) | 0 |  |
| **CNS irAE** | 9(4.4) | 62 (41-88) | 150 | 80 (42-117) | 2 (22.2) | IL-6 inhibitor (1) |
|  |  |  |  |  |  | IVIG + MMF +  cyclophosphamide (1) |
| **Other irAE** | 14(6.8) | 45 (21-419) | 60 | 29 (16-72) | 2 (14.3) | Hydrocortisone (1) |
|  |  |  |  |  |  | MMF (1) |
| ***: 11 Patients with rheumatic irAE developed chronic symptoms requiring a low (< 10 mg) maintenance dose of prednisolone. **: Patients with thyroid irAE were primarily treated with levothyroxine. ***: 3 patients received corticosteroids and hydrocortisone. irAE: immune-related adverse events, IQR: inter-quartile range, IVIG: intravenous immunoglobins, MMF: mycophenolate mofetil.** | | | | | | |
