# Supplementary figures and images for: Immune-related hepatitis and hypophysitis are associated with superior survival in melanoma patients treated with combined ipilimumab and nivolumab
Source: Oncoimmunology. 2025 Aug 8;14(1):2543510. doi: 10.1080/2162402X.2025.2543510 (PMC12952734; doi:10.1080/2162402X.2025.2543510)

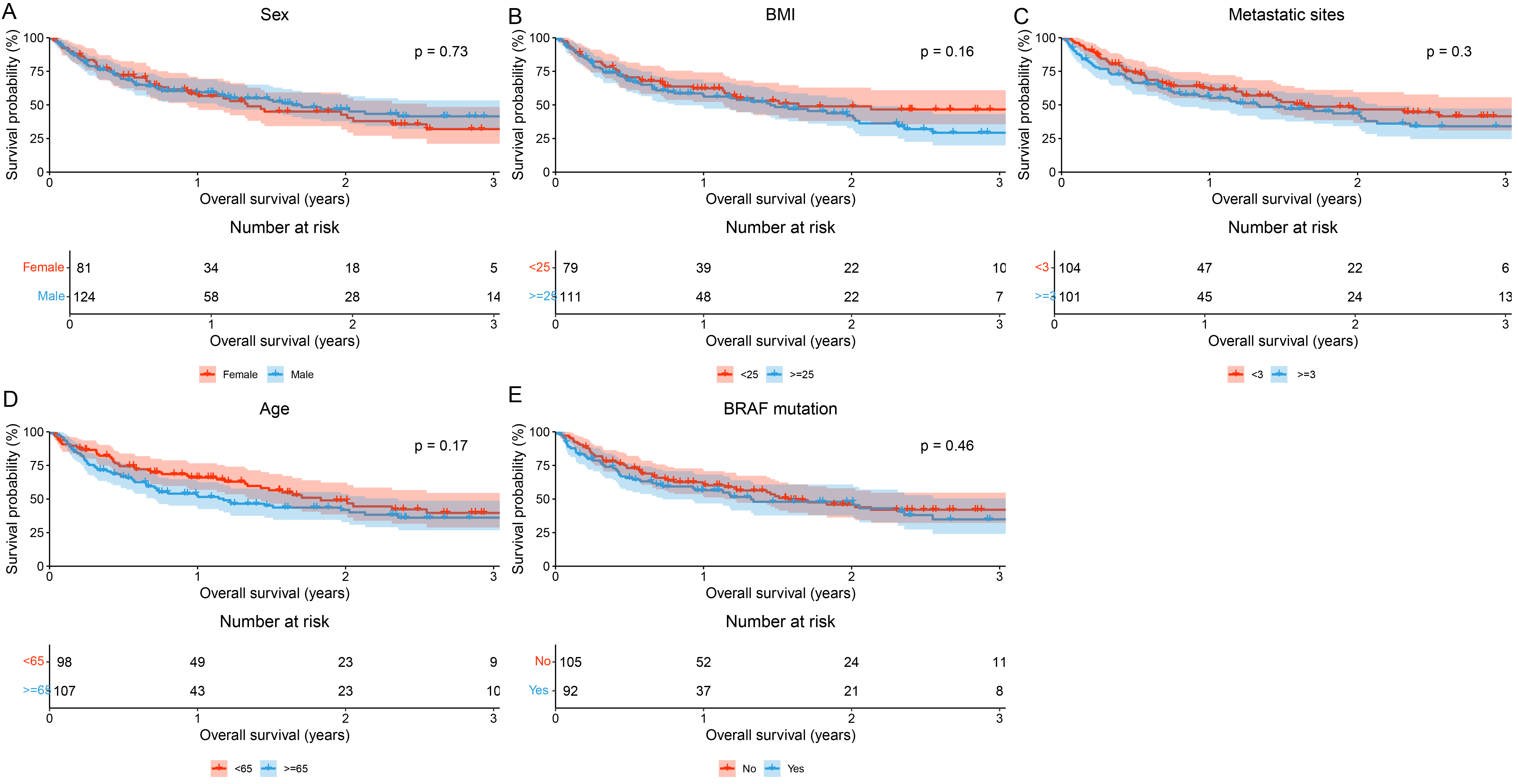

Supplement: Supplemental Material [file KONI_A_2543510_SM6212.zip › New folder/Supplementary figure S1.jpg]

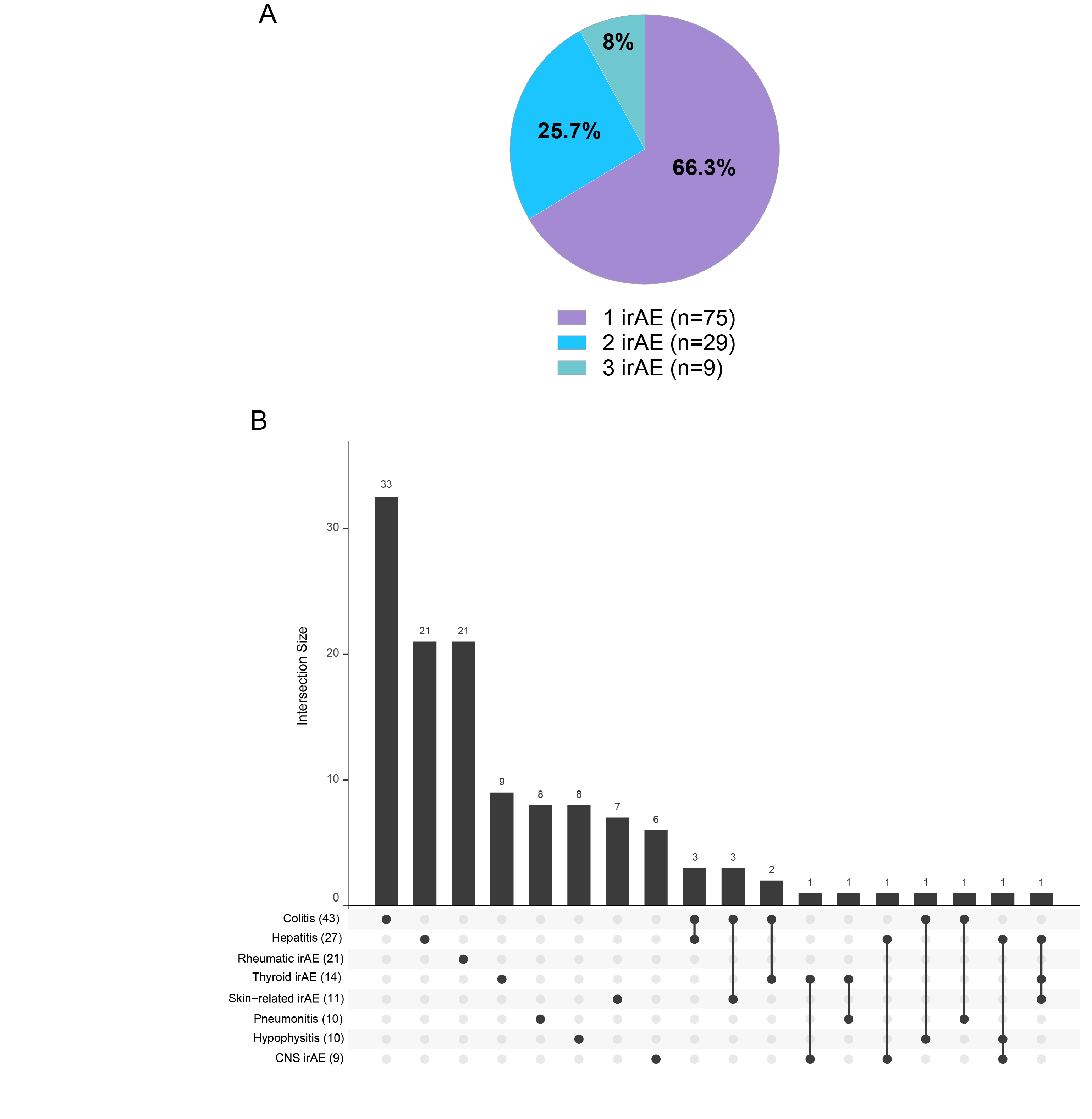

Supplement: Supplemental Material [file KONI_A_2543510_SM6212.zip › New folder/Supplementary figure S2.jpg]

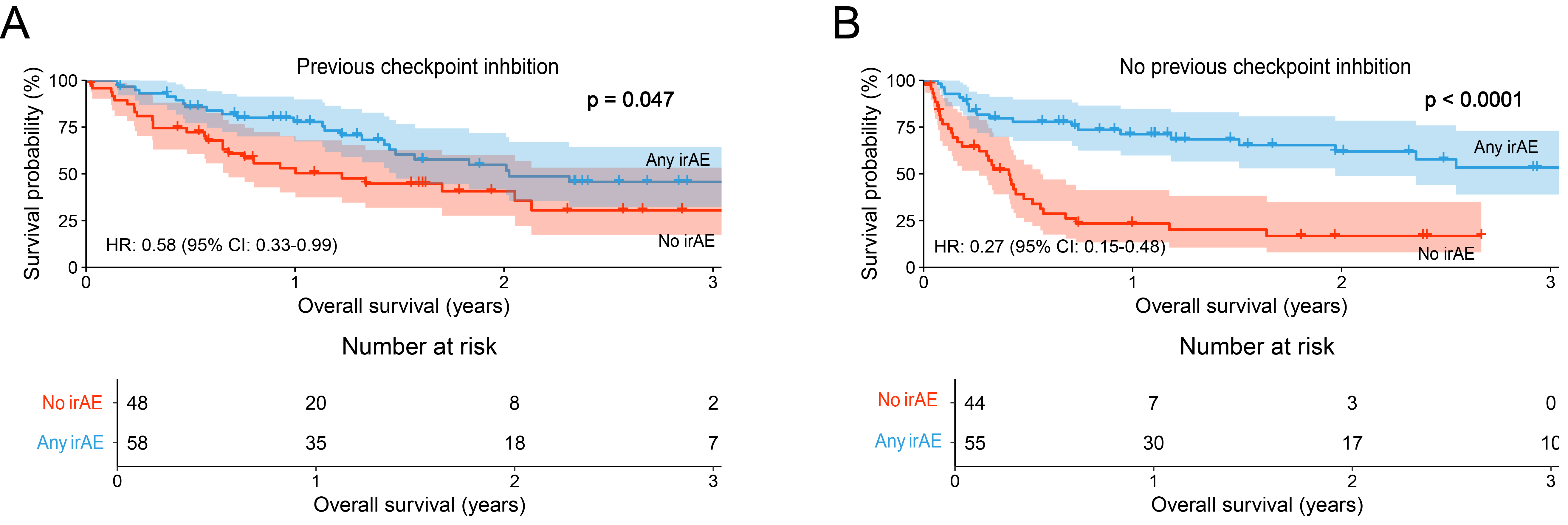

Supplement: Supplemental Material [file KONI_A_2543510_SM6212.zip › New folder/Supplementary figure S3.jpg]

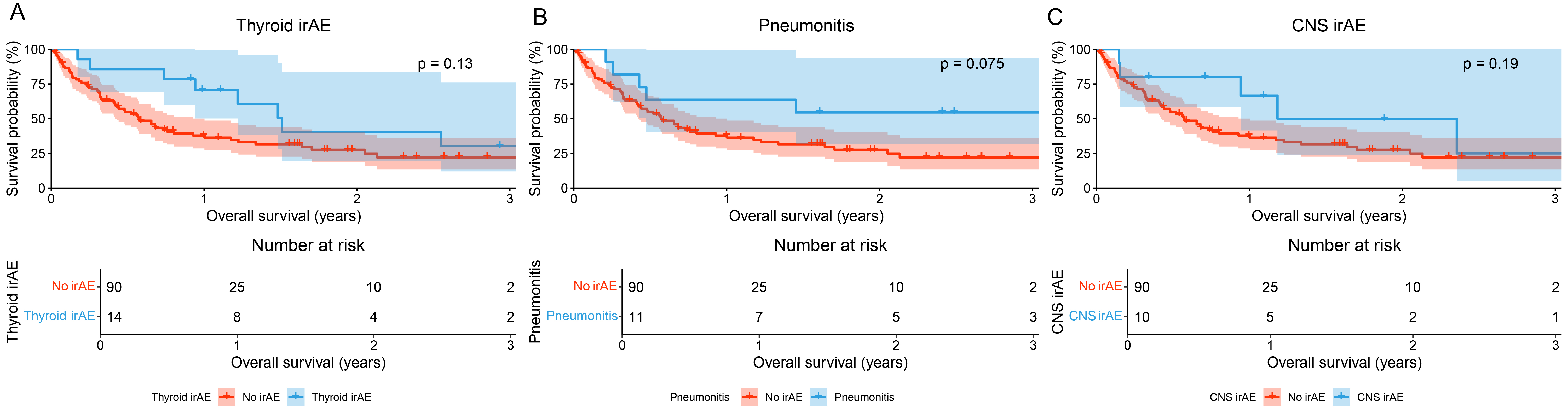

Supplement: Supplemental Material [file KONI_A_2543510_SM6212.zip › New folder/Supplementary figure S4.jpg]
